# NURSE STAFFING LEVELS AND OTHER FACTORS ASSOCIATED WITH NURSING HOME QUALITY

**DOI:** 10.1093/geroni/igae098.4033

**Published:** 2024-12-31

**Authors:** Juh Hyun shin, Charlene Harrington, Soo-Kyoung Lee, Jinhyun Ahn, Yeonwoo Lee, Sanna Ali

**Affiliations:** George Washington University, Ashburn, Virginia, United States; University of California, San Francisco, San Francisco, California, United States; Seoul National University, Seoul, Republic of Korea; Jeju National University, Jeju-si, Cheju-do, Republic of Korea; Jeju National University, Jeju-si, Cheju-do, Republic of Korea; George Washington University, WASHINGTON DC, District of Columbia, United States

## Abstract

This study evaluated the quality of nursing homes (NHs) by analyzing deficiency citations reported by the Centers for Medicare and Medicaid Services (CMS). Key factors examined included CMS nurse staffing standards, ownership types, demographic characteristics, facility size, occupancy rates, geographic location, and Medicaid funding. Data from all U.S. NHs for Fiscal Year 2019 were sourced from CMS and LTCFocus, encompassing 13,086 facilities. Using ordinary least squares (OLS) regression, the study found that NHs with a total hours per resident day (HPRD) of 4.1 and registered nurse (RN) HPRD of 0.75 experienced fewer deficiency citations (1.18 and 0.8, respectively). In contrast, the CMS 2024 standards predict 3.48 total HPRD and RN HPRD of 0.5, with deficiencies of 0.82 and 0.6. The analysis showed that higher RN, licensed nurse, and certified nursing assistant HPRD correlated with reduced deficiency scores, indicating better quality. Conversely, higher bed occupancy rates were associated with fewer deficiencies, while increased proportions of non-White residents and urban locations correlated with more deficiencies. Additionally, larger facilities and those with higher Medicaid coverage had more deficiencies. Not-for-profit NHs generally had lower deficiency scores. These results emphasize the crucial role of adequate nurse staffing in maintaining NH quality and illustrate how various provider characteristics influence deficiency levels.

